# Successful Haematopoietic Stem Cell Transplantation for LRBA Deficiency with Fludarabine, Treosulfan, and Thiotepa-Based Conditioning

**DOI:** 10.1007/s10875-024-01770-1

**Published:** 2024-09-12

**Authors:** Bella Shadur, Adeeb NasserEddin, Irina Zaidman, Yael Dinur Schejter, Ehud Even-Or, Yackov Berkun, Isabelle Meyts, Hatem Hmedat, Ashraf Sulaiman, Stuart G. Tangye, Polina Stepensky

**Affiliations:** 1 Department of Bone Marrow Transplantation and Cancer Immunotherapy, Hadassah University Medical Centre, Jerusalem, Israel; 2 Garvan Institute of Medical Research, Sydney, Australia; 3 School of Clinical Medicine, Faculty of Medicine and Health, UNSW, Sydney, NSW Australia; 4 Department of General Paediatrics, Hadassah University Medical Centre, Jerusalem, Israel; 5 Inborn Errors of Immunity, Department of Microbiology, Immunology and Transplantation, KU Leuven, Leuven, Belgium; 6 Pediatric Immunodeficiency, Department of Pediatrics, University Hospitals Leuven, Leuven, Belgium

**Keywords:** LRBA deficiency, Haematopoietic stem cell transplantation, Graft vs host disease, Infectious complications

## Abstract

LRBA deficiency is an inborn error of immunity defined by autoimmunity, lymphoproliferation, recurrent infections, cytopenia, and inflammatory bowel disease. Despite recent advances in managing this disease with targeted biologic therapy, haematopoietic stem cell transplant (HSCT) remains the only cure. However, great variability exists between protocols used to transplant patients with LRBA deficiency. We describe a cohort of seven patients with LRBA deficiency who underwent HSCT using a myeloablative, reduced toxicity regime of fludarabine, treosulfan, and thiotepa at two transplantation centres from 2016 to 2019. Data were collected both retrospectively and prospectively, measuring time to engraftment, infectious complications, incidence of graft versus host disease, and post-transplantation chimerism. Six of seven patients survived transplantation, and four of six surviving patients achieving treatment-free survival. We thus recommend that HSCT with fludarabine, treosulfan, and thiotepa-based conditioning be considered in patients with LRBA deficiency.

## Introduction

The *LRBA* (lipopolysaccharide-responsive beige-like anchor) gene was discovered in 2001 [1]. It belongs to the BEACH family of proteins and has been found to function as a chaperone for other intracellular and transmembrane proteins, protecting them from lysosomal degradation [1–5]. Biallelic deleterious variants in *LRBA* that disrupt expression and/or function of LRBA protein, resulting in a severe and often life-threatening immune dysregulatory disease, were first described in 2012 [6–8]. LRBA plays a central role in preventing lysosomal degradation of the immune regulatory protein CTLA4, thereby enabling its trafficking to the cell surface. Thus, a central physiological defect caused by impaired expression or function of LRBA protein is a secondary lack of CTLA4 expression on the surface of regulatory T (Treg) cells, leading to immune dysregulation [3]. The spectrum of disease manifestations and severity in patients with LRBA deficiency is broad, and characterised by recurrent infections, sinopulmonary disease, inflammatory bowel disease (IBD), cytopenia, and autoimmunity. Milder, and even asymptomatic cases, have also occasionally been described [3, 6–21].

A range of therapeutic options have been employed to manage the variability of clinical manifestations of LRBA deficiency. In addition to corticosteroids and steroid-sparing medications such as sirolimus [6–10, 12, 14–20, 22], CTLA4 replacement using the CTLA4-IgG4 fusion product abatacept has shown promise [3, 11, 17, 21]. However, despite evidence of successful immune modulation, cure can only be achieved by haematopoietic stem cell transplantation (HSCT) [3, 9–11, 20–23]. The first report of successful HSCT for LRBA deficiency was published in 2015 by Seidel et al., which showed that HSCT could cure all symptoms of LRBA deficiency [10]. Since then, Tesch et al. reported the largest case series to date of 24 transplanted LRBA-deficient patients, which showed that for patients with severe disease, HSCT achieves a better quality of life than conventional medical therapy or abatacept [24]. Despite these studies, no consensus exists as to the criteria for transplantation, optimal chemotherapy regimen, and degree of donor chimerism required for cure. In this report, we describe HSCT using fludarabine, treosulfan, and thiotepa-based conditioning in patients with LRBA deficiency, from two transplant centres.

## Methods

Between February 2017 and February 2019, we prospectively recruited all patients at Hadassah University Hospital diagnosed with LRBA deficiency who underwent HSCT. We documented pre-HSCT disease severity, immune deficiency and dysregulation activity (IDDA) score [24], donor HLA compatibility, graft source, chemotherapy regimen, engraftment, development of graft vs. host disease (GvHD), and infectious complications. We also retrospectively analysed transplantation data for one patient who was initially treated at Hadassah University Medical Center, but migrated to Belgium and underwent HSCT at University Hospitals Leuven. Approval for this study was obtained from the institutional review board of both institutions, in accordance with the principles of the Declaration of Helsinki, and all patients (or their parents) provided written consent for participation in this study. Statistical analysis was performed using GraphPad Prism (GraphPad Software; Boston Massachusetts).

## Results

### Pre-HSCT Patient Characteristics

Seven patients from six families were recruited over the study period, one from University Hospitals Leuven (P1) and six from Hadassah University Hospital (P2-7). All patients were of Palestinian Arab ethnic origin and of consanguineous parentage. Baseline patient information is presented in Table 1. Index cases were diagnosed via whole exome or Sanger sequencing (for siblings of index cases), and a lack of expression of LRBA protein was confirmed via Western Blot for all patients. The average patient IDDA score prior to HSCT was 41.5 (range 15–78), and patients exhibited a range of disease manifestations, particularly recurrent infections, cytopenia, lymphadenopathy, hepatosplenomegaly, sinopulmonary disease (including bronchiectasis), and inflammatory bowel disease (Table 1). Transplantation was undertaken when medical therapy failed to control life-threatening disease manifestations (P4, 5, and 6), or the cumulative disease burden was deemed too high by the patient and/or family (P1, 2, 3, and 7). For the first group (P4-6), HSCT was undertaken urgently, whereas for the latter (P1-3, P7) group the decision to proceed to transplantation, and the timing of transplantation, was individualized.

**Table 1:** Pre-HSCT patient characteristics

Patient	1	2	3	4	5	6	7
Gene variant	c.7970T > G, p.I2657S	c.7970T > G, p.I2657S	c.7869_7873delTTCTA, p.S2624Rfs*23	c.8437 C > T, pQ2813X	c.7869_7873delTTCTA, p.S2624Rfs*23	c.8174_8175insCATG, p.N2727Hfs*13	c.7970T > G, p.I2657S
Gender	F	M	M	M	M	M	M
Ethnicity	Palestinian Arab						
Age at diagnosis	9	14	26	2	8	9	3
Disease manifestations	Cytopenia Lymphadenopathy Splenomegaly HG Bronchiectasis Recurrent infections Failure to thrive	Cytopenia Lymphadenopathy Hepatosplenomegaly IBD HG Bronchiectasis Recurrent infections Vitiligo Failure to thrive	Cytopenia Lymphadenopathy Hepatomegaly IBD HG Bronchiectasis Emphysematous lung disease Recurrent infections Neurological features	Severe IBD with metabolic acidosis and electrolyte disturbance Lymphadenopathy Pneumonia Recurrent infections Failure to thrive	IBD Recurrent infections Splenomegaly	Cytopenia Lymphadenopathy HG Sinopulmonary disease Recurrent infections	Lymphadenopathy IBD HG Bronchiectasis Recurrent infection Dysuria Failure to thrive
Pre-HSCT Treatment	IVIg Hydroxychloroquine Abatacept	IVIg Prednisolone Mycophenolate mofetil Sirolimus	IVIg Prednisolone Rituximab Foscarnet for CMV infection	Prednisolone Sirolimus Methotrexate	Prednisolone Sirolimus	IVIg Prednisolone Mycophenolate mofetil Sirolimus Abatacept	IVIg Abatacept
Pre-HSCT IDDA Score	38	36	55	78	33	15	36
Indication for transplant	Cumulative disease burden	Cumulating disease burden	Cumulative disease burden	Life-threatening, treatment-refractory IBD with electrolyte disturbance	Life threatening IBD	Treatment-refractory thrombocytopenia	Cumulative disease burden

F: female; M: male, HG: hypogammaglobulinemia; IVIg: intravenous immunoglobulin; IBD: inflammatory bowel disease; CMV: cytomegalovirus

### HSCT and Donor Characteristics

HSCT and graft characteristics are summarised in Table 2. The median age at transplant was 9 years (range 3–25 years). Patients were transplanted with a reduced toxicity conditioning regimen. P1 underwent HSCT at University Hospitals Leuven and received fludarabine and treosulfan conditioning, with alemtuzumab serotherapy as per protocol D of the EBMT/ESID inborn errors working party guidelines [25]. All other patients underwent HSCT at Hadassah University Medical Center and received rituximab 10 days prior to graft infusion, and underwent conditioning with fludarabine, treosulfan, and thiotepa, with anti-thymocyte globulin (ATG) serotherapy as per protocol B of the EBMT/ESID guidelines [25]. All patients were transplanted using bone marrow derived stem cells, five from matched sibling donors, one from a matched related donor, and one from a 9/10 matched unrelated donor. Of the related donors, two were wild type for *LRBA* and four were heterozygous carriers of a pathogenic *LRBA* allele (Table 2). Cyclosporine A (CsA) and mycophenolate mofetil (MMF) were used for GvHD prophylaxis in both centres. HSCT was performed in high efficiency particulate absorbing (HEPA) filtered negative pressure rooms, with anti-microbial prophylaxis and virus screening (CMV, EBV, adenovirus, HHV6) as per local guidelines. Whole-blood chimerism was assessed initially at day + 30 post-HSCT, and then at three-monthly intervals to one year post-transplant, and yearly thereafter.

**Table 2:** HSCT characteristics and outcomes

Patient	1	2	3	4	5	6	7
Age at transplant (years)	9	17	26	3	8	13	5
Transplant centre	University Hospital Leuven	Hadassah University Medical Center					
Conditioning regimen	Fludarabine 30mg/m^2^, d-9 to -5 Treosulfan 14 g/m^2^, d-8 to -6	Rituximab 375mg/m^2^, day − 10; Fludarabine 30mg/m^2^, d-9 to -5; Treosulfan 14 g/m^2^, d-8 to -6; Thiotepa 5 mg/kg, d-4 to -3					
GvHD Prophylaxis	Alemtuzumab 0.2 mg/kg, day − 8 to -4 MMF and CsA	ATG (Patients P2-P6 received Thymoglobulin 2.5 mg/kg, d-4 to d-1; P7 received Fresenius 15 mg/kg, d-4 to d-2) CsA 3 mg/kg, from d-1; MMF 15 mg/kg three times per day, from d + 1 to d + 30					
Donor source	MSD (WT)	MRD (HZ)	MSD (HZ)	MSD (HZ)	9/10 MUD	MSD (WT)	MSD (HZ)
Cell source	Bone marrow	Bone marrow	Bone marrow	Bone marrow	Bone marrow	Bone marrow	Bone marrow
Cell dose (CD34 × 10^6^/kg)	3.27	3.46	2.53	1.35	2.53	1.9	4.66
Engraftment (days) Neutrophils/platelets	30/31	22/22	-	19/18	19/29	19/22	20/32
G-CSF doses	0	5	7	0	5	2	0
Transfusions (RBC/platelets)	Information not available	8/35	9/78	1/5	16/35	8/8	3/13
Infections	Parainfluenza virus Adenovirus reactivation CMV reactivation Staphylococcus epidermidis sepsis	Aspergillus AOM Fungal lung cavitation EBV liver and viremia CMV colitis BK virus with haemorrhagic cystitis	Klebsiella	Nil	Recurrent adenovirus colitis and viremia BK virus with haemorrhagic cystitis CMV colitis	BK virus with haemorrhagic cystitis CMV, HHV-6, and adenovirus viremia	CMV viraemia Salmonella diarrhoea
Acute GvHD	No	Skin: grade 1 Gut: grade 3 (treated with steroids & ECP)	-	Skin, grade 2	Gut, grade 4	No	Skin, grade 2
Chronic GvHD	Scleroderma Chronic kidney disease	No	-	No	No	Yes	No
Other complications	ITP- treated with Rituximab Unconditioned CD34^+^ top-up (4 × 10^6^/kg) four months post- transplant	Neutropenia day + 60 (treated with G-CSF) Oral ulcers	Severe mucositis Gram negative sepsis Respiratory failure Atrial fibrillation Hypotension Multi-organ failure ARDS	Donor chimerism 96% day + 30, 92% at six months Treated with 1 × 10^6^ cells/kg DLI	Peripheral neuropathy Prolonged post-HSCT diarrhoea for one year	Grade IV GvHD of the liver following 10 × 10^6^/kg DLI at eight months post-HSCT (given to treat donor chimerism of 84%) Gastric ulcers and melena	Nil
Donor chimerism	Donor	Donor	-	74%	Donor	Donor	Donor
Outcome, years post-transplant	Alive, 5 IVIg every two months	Alive, 5 ITP resolved with steroid Recent episode of pneumococcal pneumonia	Deceased, day + 13	Alive, 4 Treatment free	Alive, 4	Alive, 4 Treatment free	Alive, 4 Treatment free
Post-HSCT IDDA score	11.5	3.8	N/A	0	0	0	3

MMF: Mycophenolate mofetil; CsA: Cyclosporine A; ATG: Antithymocyte globulin; MSD: matched sibling donor; MRD: matched related donor; MUD: matched unrelated donor; HZ: heterozygous (carrier); WT: wild type; G-CSF: granulocyte colony stimulating factor; GvHD: graft vs. host disease; ITP: immune thrombocytic purpura; RBC: red blood cells; AOM: Acute Otitis Media, EBV: Epstein Barr Virus; DLI: donor lymphocyte infusion; ARDS: Acute Respiratory Distress Syndrome, DLI: Donor Lymphocyte Infusion

### Transplant Outcomes

Transplant outcomes are summarised in Table 2. Six patients survived HSCT, yielding an overall survival (OS) rate of 85.7%, with five of six surviving patients demonstrating full donor chimerism at an average of four years post-HSCT. Three of six surviving patients are living symptom- and treatment-free, and P1 (with ongoing chronic GvHD (cGvHD)) is able to participate in all acts of daily living. IDDA score decreased from a pre-transplant mean of 41.5 to a post-transplant mean of 3.05 (range: 0-11.5). Use of heterozygous or wild type donors did not impact transplant outcome, irrespective of how this was assessed or measured.

Neutrophil engraftment (defined as a neutrophil count ≥ 0.5 × 10^9^ cells/L for three consecutive days) occurred on average at day + 21.5 post-HSCT. Platelet engraftment (defined as a platelet count ≥ 20 × 10^9^ cells/L for three consecutive days), took longer at a mean of 25.7 days post-HSCT. Patients required greater platelet support than they did red blood cell support, with the average number of platelet transfusions by day 100 standing at 29 compared to the average number of blood unit transfusions being 7.5 (Table 2).

Three patients developed acute GvHD of the skin, which was mild in all cases and resolved with topical steroid therapy. In contrast, two patients (P2 and P5) developed severe GVHD of the gut (grade III and grade IV disease, respectively), which in both cases was associated with viral reactivation, making differentiation between GvHD and viral-induced colitis difficult. In P2, grade III GvHD was associated with EBV hepatitis and CMV colitis. Consequently, P2 required treatment with rituximab, foscarnet, steroids, and extra-corporeal photophoresis (ECP) for one month. P5 had suffered pre-HSCT CMV colitis, EBV colitis, and was known to carry adenovirus and HHV6 in his stool prior to transplantation. Pre-HSCT diarrhoea continued and three weeks post-transplantation he suffered reactivation of CMV colitis which was treated with ganciclovir. At 10 weeks post-HSCT he developed adenovirus colitis which was treated with cidofovir. Diarrhoea in P5 Itook approximately one year to completely settle. In contrast to P2 and P5, P4 suffered severe, life-threatening diarrhoea prior to transplant which completely resolved with his first dose of ATG; he suffered no post-transplant gastrointestinal GvHD despite undergoing donor lymphocyte infusion (DLI) six months post-HSCT to try and boost donor chimerism.

Two patients developed cGvHD. P1 suffers ongoing cGvHD in the form of scleroderma and kidney disease. She is non-compliant with treatment, receiving IVIg approximately every eight weeks and not taking her prescribed Imatinib. Despite this, she has good quality of life and is not limited in activities of daily living. P6 developed grade IV GvHD of the liver at day + 270 post-transplant, following a 10 × 10^6^ lymphocytes/kg DLI that was given due to concerns that falling donor chimerism (84% on whole blood assessment at the time of DLI) would lead to reappearance of disease features secondary to immune dysregulation in the patient’s returning immune system. Liver biopsy also found hepatic adenovirus, parvovirus, and HHV-6. Bilirubin peaked at 542µmol/L (normal range: 0–17 µmol/L), with direct bilirubin at 484µmol/L (normal range 0-3.4 µmol/L). Various therapies were trialled including steroids, tacrolimus, Etanercept, CsA, sirolimus, IVIg, ruxolitinib, IL2, and ECP with limited success. Eventually, liver GvHD was brought under control with Alemtuzumab. P6 is currently three years post-initial HSCT, with full donor chimerism, normal liver function, and is treatment-free (Table 2).

The only fatality was P3, who was the oldest patient in our cohort and had accumulated the most comorbidities prior to HSCT (IDDA: 55, Table 1), with particularly severe lung disease characterised by widespread bronchiectasis and emphysematous lung changes. He rapidly developed multi-organ failure and died 13 days after transplant.

## Discussion

The first individual case report of successful HSCT for LRBA was published in 2015 [10], with the first case series collating data from 12 patients who underwent HSCT for LRBA deficiency following in 2018 [22]. It was reported that four patients achieved complete treatment-free remission, four patients achieved partial remission and required ongoing medical therapy, and four patients died secondary to transplant-related complications. Thus, the OS in this study was 67%. These publications established, crucially, that (a) LRBA deficiency was amenable to transplantation, (b) non-haematological aspects of the disease were cured by HSCT, and (c) heterozygous family members could serve as donors for HSCT [9–11, 20, 22, 23].

These 12 cases were subsequently included in the largest comparison between medical therapy and HSCT in LBRA defiiciency, published in 2020, which also included our cohort of patients [24]. Tesch et al. reported that the OS for the 52 patients who received medical therapy (including abatacept) was higher than that of the 24 transplanted patients (82.7% vs. 70.8%, respectively). However, transplanted patients had a significantly higher pre-transplantation disease activity score than those who continued to receive medical therapy (IDDA: pre 32.9 vs. post 20.8, *p* = 0.006), and patients transplanted after 2015 (*n* = 10) recorded an OS of 90%, compared to 57.1% OS for patients undergoing HSCT prior to 2015 (*n* = 14). This suggests that transplant data is skewed by early experiences with patients who had more severe disease and underwent HSCT late in their disease and treatment course, and that outcomes improved with time and familiarity with both the disease and transplantation for LRBA deficiency. Indeed, patients transplanted within three years of diagnosis with low disease burden demonstrated 100% OS. Lung disease was a particularly severe risk factor, as all transplant-related deaths occurred in those with lung disease (i.e. OS was 100% in patients with no pulmonary manifestations of LRBA deficiency). Most promisingly, patients who survived HSCT reported significantly lower IDDA scores than those who remained untransplanted (6.3 vs. 20.8, respectively; *p* = 0.005), suggesting that timely transplant can prevent years of burdensome disease [24].

Whilst these cohorts provided crucial evidence regarding the promise of HSCT, certain questions remain unanswered. Specifically, conditioning regimens varied widely between transplant centres, with some employing myeloablative conditioning (MAC) and others choosing various reduced intensity or sub-myeloablative or reduced toxicity combinations [3, 9–11, 20–23]. There remains no consensus as to the minimal level of acceptable post-transplantation donor chimerism, and the use of bone marrow vs. peripherally-derived donor stem cells has not been addressed. In our cohort, we describe seven patients with LRBA deficiency who underwent HSCT with a reduced toxicity regimen of fludarabine and treosulfan, with the addition of thiotepa and pre-conditioning rituximab in six of seven patients. In patients P2-P7, thiotepa was added as it is the experience of the treating centre that this significantly increases the likelihood of achieving full donor chimerism [26], which is important given the immune dysregulation often associated with LRBA deficiency. In addition to chemotherapy, all patients received serotherapy even though five of seven patients underwent transplantation using a MSD. There is evidence from the oncology literature that, in the setting of MSD transplant, the addition of serotherapy reduces the risk of GvHD without negatively impacting survival and relapse rate [27–29]. We would hypothesize that in non-malignant immune deficiency disorders with significant immune dysregulation, the addition of serotherapy would also decrease the rate of GvHD.

OS in our study was 85.7%, in-line with that described by Tesch et al. for patients transplanted after 2015 [24]. The one fatality in our cohort was the oldest patient who had accumulated the most comorbidities and pre-HSCT risk factors, making his transplant very high risk: severe bronchiectasis, emphysematous lung disease, splenectomy, and colectomy. All surviving patients are in complete disease remission and all but one demonstrate full donor chimerism: P4’s last recorded whole-blood chimerism was 74% at 15-months post-HSCT. We have not been able to assess individual lineage chimerism, as it is not performed at the treating centre, thus it is possible his T cell (and Treg) chimerism is higher than that of B and myeloid cell lineages, and is sufficiently controlling immune dysregulation. As he remains treatment- and symptom-free, we have not pursued any further lymphocyte or stem-cell infusions to try and improve his whole blood chimerism. We aimed for full donor chimerism in all patients, although P6 demonstrates the risk of DLI to achieve this, and P4’s successful outcome suggests full donor chimerism may not be necessary although longer-term monitoring will be necessary to answer this question.

GvHD caused significant burden in our cohort and whilst cutaneous GvHD was generally mild, gut GvHD was particularly severe in several patients. This likely reflects significant pre-HSCT gastrointestinal viral load, a pre-existing inflammatory milieu, and prolonged engraftment time in the gastrointestinal tract post-HSCT. P1 is the only patient with ongoing cGvHD, however they can perform all acts of daily living with good quality of life despite ongoing medical therapy. Interestingly, no patient who received pre-HSCT rituximab developed cGvHD (with the exception of P6, in whom hepatic cGvHD was related to DLI). Whilst rituximab is not routinely included in HSCT conditioning regimens, there is some evidence of its efficacy in preventing acute and chronic GvHD in transplantation undertaken to cure malignant disease [30–34]. Despite our small sample size, these results show promise and the role of prophylactic rituximab in primary immune disorders with a significant immune dysregulatory component warrants further investigation, as does the role of serotherapy in patients with immune dysregulatory and/or immune deficiency diseases.

Despite the overall success of HSCT in our cohort, the path to cure was arduous for most patients. Prolonged time to engraftment of neutrophils and platelets meant patients suffered a high burden of infectious complications and required significant supportive therapy with multiple and prolonged courses of anti-microbials, and prolonged blood product support. Viral reactivation was seen in five of six surviving patients. All patients in our cohort were transplanted with marrow-derived stem cells, and it is possible that the use of peripheral blood-derived stem cells would lead to faster engraftment and lessen the infectious burden, albeit at the cost of an increased risk of GvHD. It is also likely that earlier progression to HSCT would decrease the pre-transplantation infectious burden.

Three patients in our cohort (P1, P6, and P7) were treated with abatacept prior to transplantation (P1: two months; P6 and P7: six months). All three suffered varying degrees of pulmonary compromise prior to transplantation, and abatacept was introduced to ameliorate lung disease and other clinical manifestations (e.g. severe thrombocytopenia in P6), as well as to generally optimise their clinical condition prior to transplantation. In P1 and P7, abatacept treatment led to improved CT appearance of their lungs. It is interesting to compare their post-transplant outcomes to those of P3 who did not receive abatacept therapy. Severe lung disease is a risk factor for transplantation in LRBA deficiency and other primary immune deficiencies [24], however abatacept has been shown to dramatically improve lung function in LRBA deficient patients [3]. However, given the severity of P3’s lung disease and his many comorbidities, it is unlikely that prolonged therapy with abatacept would have changed P3’s transplant outcome. Of note, a recent study demonstrated similar efficacy of abatacept and HSCT in LRBA deficient patients, particularly if abatacept was introduced prior to onset of severe disease [35]. This suggests that long-term abatacept therapy may be an alternative to HSCT in some patients, however the lack of long-term data as well as the high cost and poor availability of abatacept in many centres means this will not be feasible for all patients. Other studies have noted improvement of some disease features (e.g. lymphoproliferation) and not others (e.g. ITP) [21]. We advocate considering the use of abatacept in patients with LRBA deficiency prior to HSCT, as part of optimising their clinical condition and acting as a bridge to transplantation [35]. The timing of abatacept cessation prior to HSCT remains an open question: P6 and P7 received monthly intravenous abatacept and received their last infusion approximately one month prior to transplantation, whereas P1 ceased abatacept several months prior to HSCT as they migrated to Belgium and received no further doses.

## Conclusion

Our findings reinforce previously published findings that HSCT leads to excellent clinical outcomes in LRBA deficiency. Despite its challenges, cure of LRBA deficiency is possible by HSCT and remains the best long-term treatment option for patients with a high burden of disease. As familiarity with transplanting LRBA deficiency increases and patients undergo transplantation earlier and with a lower disease burden, together with improved donor matching and increased use of serotherapy, our expectation is that complications such as viral reactivation and severity of GvHD will decline. Optimal timing of transplantation remains an open question, as does the degree of donor chimerism required for sustained cure. However, our data establish that cure of LRBA deficiency is possible with a conditioning regimen based on fludarabine, treosulfan, and thiotepa.

## Data Availability

No datasets were generated or analysed during the current study.

## References

[1] Wang J-W, Howson J, Haller E, Kerr WG. Identification of a novel lipopolysaccharide-inducible gene with key features of both a kinase anchor proteins and chs1/beige proteins. J Immunol. 2001;166:4586–95. 11254716 10.4049/jimmunol.166.7.4586

[2] Cullinane AR, Schaffer AA, Huizing M. The BEACH is hot: a LYST of emerging roles for BEACH-domain containing proteins in human disease. Traffic. 2013;14(7):749–66. 23521701 10.1111/tra.12069 PMC3761935

[3] Lo B, Zhang K, Lu W, Zheng L, Zhang Q, Kanellopoulou C, et al. Patients with LRBA deficiency show CTLA4 loss and immune dysregulation responsive to abatacept therapy. Science. 2015;349(6246):436–40. 26206937 10.1126/science.aaa1663

[4] Wang J-W, Gamsby JJ, Highfill SL, Mora LB, Bloom GC, Yeatman TJ, et al. Deregulated expression of LRBA facilitates cancer cell growth. Oncogene. 2004;23(23):4089–97. 15064745 10.1038/sj.onc.1207567

[5] Gebauer D, Li J, Jogl G, Shen Y, Myszka DG, Tong L. Crystal structure of the PH-BEACH domains of human LRBA/BGL. Biochemistry. 2004;43(47):14873–80. 15554694 10.1021/bi049498y

[6] Lopez-Herrera G, Tampella G, Pan-Hammarstrom Q, Herholz P, Trujillo-Vargas CM, Phadwal K, et al. Deleterious mutations in LRBA are associated with a syndrome of immune deficiency and autoimmunity. Am J Hum Genet. 2012;90:986–1001. 22608502 10.1016/j.ajhg.2012.04.015 PMC3370280

[7] Alangari A, Alsultan A, Adly N, Massaad MJ, Kiani IS, Aljbereen A, et al. LPS-responsive beige-like anchor (LRBA) gene mutation in a family with inflammatory bowel disease and combined immunodeficiency. J Allergy Clin Immunol. 2012;130:481–8. 22721650 10.1016/j.jaci.2012.05.043 PMC3582381

[8] Burns SO, Zenner HL, Plagnol V, Curtis J, Mok K, Eisenhut M, et al. *LRBA* gene deletion ina patient presenting with autoimmunity without hypogammaglobulinemia. J Allergy Clin Immunol. 2012;130(6):1428–32. 22981790 10.1016/j.jaci.2012.07.035 PMC3930010

[9] Tesi B, Priftakis P, Lindgren F, Chiang SC, Kartalis N, Lofstedt A, et al. Successful hematopoietic stem cell transplantation in a patient with LPS-responsive beige-like anchor (LRBA) gene mutation. J Clin Immunol. 2016;36:480–9. 27146671 10.1007/s10875-016-0289-y

[10] Seidel MG, Hirschmugl T, Gamez-Diaz L, Schwinger W, Serwas N, Deutschmann A, et al. Long-term remission after allogeneic hematopoietic stem cell transplantation in LPS-responsive beige-like anchor (LRBA) deficiency. J Allergy Clin Immunol. 2015;135(5):1384–e901. 25539626 10.1016/j.jaci.2014.10.048 PMC4429722

[11] Gamez-Diaz L, August D, Stepensky P, Revel-Vilk S, Seidel MG, Noriko M, et al. The extended phenotype of LPS-responsive beige-like anchor protein (LRBA) deficiency. J Allergy Clin Immunol. 2016;137:223–30. 26768763 10.1016/j.jaci.2015.09.025

[12] Serwas NK, Kansu A, Santos-Valente E, Kuloglu Z, Demir A, Yaman A, et al. Atypical manifestation of LRBA deficiency with predominant IBD-like phenotype. Inflamm Bowel Dis. 2015;21(1):40–7. 25479458 10.1097/MIB.0000000000000266

[13] Charbonnier L-M, Janssen E, Chou J, Ohsumi TK, Keles S, Hsu JT, et al. Regulatory T cell deficiency and immune dysregulation, polyendocrinopathy, enteropathy, X-linked-like disorder due to loss of function mutations in LRBA. J Allergy Clin Immunol. 2015;135(1):217–27. 25468195 10.1016/j.jaci.2014.10.019 PMC4289093

[14] Revel-Vilk S, Fischer U, Keller B, Nabhani S, Gamez-Diaz L, Rensing-Ehl A, et al. Autoimmune lymphoproliferative syndrome-like disease in patients with *LRBA* mutation. Clin Immunol. 2015;159(1):84–92. 25931386 10.1016/j.clim.2015.04.007

[15] Alkhairy OK, Abolhassani H, Rezaei N, Fang M, Anderson KK, Chavoshzadeh Z, et al. Spectrum of phenotypes Associated with mutations in *LRBA*. J Clin Immunol. 2016;36(1):33–45. 26707784 10.1007/s10875-015-0224-7

[16] Schreiner F, Plamper M, Dueker G, Schoenberger S, Gamez-Diaz L, Grimbacher B, et al. Infancy-onset T1DM, short stature, and severe immunodysregulation in two siblings with a homozygous *LRBA* mutation. J Clin Endocrinol Metab. 2016;101(3):898–904. 26745254 10.1210/jc.2015-3382

[17] Levy E, Stolzenberg M-C, Bruneau J, Breton S, Neven B, Sauvion S, et al. LRBA Deficiency with autoimmunity and early onset chronic erosive polyarthritis. J Clin Immunol. 2016;168:88–93. 10.1016/j.clim.2016.03.006 27057999

[18] Bakhtiar S, Ruemmele F, Charbit-Henrion F, Levy E, Rieux-Laucat F, Cerf-Bensussan N et al. Atypical manifestation of LPS-responsive beige-like anchor deficiency syndrome as an autoimmune endorine disorder with enteropathy and immunodeficiency. Front Pediatr. 2016;4(98). 10.3389/fped.2016.00098 PMC5022363 27683652

[19] Shokri S, Nabavi M, Hirschmugl T, Aghamohammadi A, Arshi S, Bemanian MH, et al. LPS-responsive beige-like anchor gene mutation associated with possible bronchiolitis obliternas organizing pneumonia associated with hypogammaglobulinemia and normal IgM phenotype and low numbers of B cells. Acta Med Iran. 2016;54(10):620–3. 27888588

[20] Sari S, Dogu F, Hwa V, Haskologlu S, Dauber A, Rosenfeld R, et al. A successful HSCT in a girl with novel LRBA mutation with refractiry celiac disease. J Clin Immunol. 2016;36(1):8–11. 26686526 10.1007/s10875-015-0220-y PMC7254980

[21] Kostel Bal S, Haskologlu S, Serwas NK, Islamoglu C, Aytekin C, Kendirli T, et al. Multiple presentations of LRBA deficiency: a single-center experience. J Clin Immunol. 2017;37(8):790–800. 28956255 10.1007/s10875-017-0446-y PMC7086713

[22] Seidel MG, Bohm K, Dogu F, Worth AJ, Thrasher A, Florkin B, et al. Treatment of severe forms of LPS-responsive beige-like anchor protein deficiency with allogeneic hematopoietic stem cell transplantation. J Allergy Clin Immunol. 2018;141(2):770–5. 28502825 10.1016/j.jaci.2017.04.023

[23] Bakhtiar S, Gamez-Diaz L, Jarisch A, Soerensen J, Grimbacher B, Belohradsky B, et al. Treatment of infantile inflammatory bowel disease and autoimmunity by allogeneic stem cell transplantation in LPS-responsive beige-like anchor deficiency. Front Immunol. 2017;8:52. 28197149 10.3389/fimmu.2017.00052 PMC5281554

[24] Tesch VK, Abolhassani H, Shadur B, Zobel J, Mareika Y, Sharapova S, et al. Long-term outcome of LRBA deficiency in 76 patients after various treatment modalities as evaluated by the immune deficiency and dysregulation activity (IDDA) score. J Allergy Clin Immunol. 2020;145(5):1452–63. 31887391 10.1016/j.jaci.2019.12.896

[25] Lankester AC, Albert MH, Booth C, Gennery AR, Gungor T, Honig M, et al. EBMT/ESID inborn errors working party guidelines for hematopoietic stem cell transplantation for inborn errors of immunity. Bone Marrow Transpl. 2021;56(9):2052–62. 10.1038/s41409-021-01378-8 PMC8410590 34226669

[26] Dinur-Schejter Y, Krauss AC, Erlich O, Gorelik N, Yahel A, Porat I, et al. Bone marrow transplantation for non-malignant diseases using treosulfan-based conditioning. Pediatr Blood Cancer. 2015;62(2):299–304. 25284797 10.1002/pbc.25267

[27] Kroger N, Solano C, Wolschke C, Bandiini G, Patriarca F, Pini M, et al. Antilymphocyte globulin for prevention of chronic graft-versus-host disease. NEJM. 2016;374(1):43–53. 26735993 10.1056/NEJMoa1506002

[28] Chang Y, Wu D, Lai Y, Q L, Sun Y, Hu J, et al. Antithymocyte globulin for matched sibling donor transplantation in patients with hematologic malignancies: a multicenter, open-label, randomized controlled study. J Clin Oncol. 2020;38(29):3368–76. 10.1200/JCO.20.00150 32650683

[29] Bacigalupo A. ATG in allogeneic stem cell transplantation: standard of care in 2017? Point. Blood Adv. 2017;1(9):569–72. 29296976 10.1182/bloodadvances.2016001560 PMC5728596

[30] Ratanatharathorn V, Logan BR, Wang D, Horowitz M, Uberti JP, Ringden O, et al. Prior Rituximab correlates with less acute graft-versus-host disease and better survival in B-cell lymphoma patients who received allogeneic peripheral blood stem cell transplantaion (PBSCT). Br J Aematol. 2009;145(6):816–24. 10.1111/j.1365-2141.2009.07674.x PMC3355660 19344418

[31] Solomon SR, Sizemore CA, Ridgeway M, Zhang X, Borwn S, Holland HK, et al. Safety and efficacy of rituximab-based first line treatment of chronic GVHD. Bone Marrow Transpl. 2019;54:1218–26. 10.1038/s41409-018-0399-7 30518977

[32] Liu L, Zhang Y, Liu S, Zhou H, Wang Q, Qiu H, et al. Outcomes of conditioning with rabbit antithymocyte globulin and rituximab in haploidentical haematopoietic stem cell transplantation in patients with severe aplastic anaemia. Bone Marrow Transpl. 2020;55:1854–6. 10.1038/s41409-020-0788-6 31959891

[33] Nakasone H, Sahaf B, Miklos DB. Therapeutic benefits targeting B-cells in chronic graft-versus-host disease. Int J Hematol. 2015;101:438–51. 25812839 10.1007/s12185-015-1782-4

[34] Cutler C, Kim HT, Bindra B, Sarantopoulos S, Ho VT, Chen YB, et al. Rituximab prophylaxis prevents corticosteroid-requiring chronic GVHD after allogeneic peripheral blood stem cell transplantation: results of a phase 2 trial. Blood. 2013;122(8):1510–7. 23861248 10.1182/blood-2013-04-495895 PMC3750344

[35] Taghizade N, Babayeva R, Kara A, Karakus IS, Catak MC, Bulutoglu A, et al. Therapeutic modalities and clinical outcomes in a large cohort with LRBA deficiency and CTLA4 insufficiency. J Allergy Clin Immunol. 2023;152(6):1634–45. 37595759 10.1016/j.jaci.2023.08.004

